# The Impact of Gratifications on Fake News Sharing Among Chinese Social Media Users and Its Mechanisms

**DOI:** 10.3390/bs16071112

**Published:** 2026-07-03

**Authors:** Yang Shao, Xuying Wang, Zhibin Jiao, Yongjie Li, Hua Jin

**Affiliations:** 1 Key Research Base of Humanities and Social Sciences of the Ministry of Education, Academy of Psychology and Behavior, Tianjin Normal University, Tianjin 300387, China; 2410340016@stu.tjnu.edu.cn (Y.S.); wangxuying@stu.tjnu.edu.cn (X.W.); jzbpsy@stu.tjnu.edu.cn (Z.J.); 2 Faculty of Psychology, Tianjin Normal University, Tianjin 300387, China; 3 Business School, Inner Mongolia University of Finance and Economics, Huhhot 010070, China

**Keywords:** fake news sharing, PLS-SEM, fsQCA, Uses and Gratifications theory, Stimulus–Organism–Response

## Abstract

The literature pays little attention to the impact of gratifications on fake news sharing among Chinese social media users, and fuzzy-set qualitative comparative analysis (fsQCA) is rarely used in fake news research. This study investigated the relationship between gratifications and fake news sharing among Chinese users. Study 1 employed partial least squares structural equation modeling (PLS-SEM) and fsQCA on the questionnaire data from 315 participants. Study 2 analyzed predictions of self-reported sharing intentions in task scenarios using data from 98 new participants. The PLS-SEM revealed that instant news sharing was positively predicted by time-passing, entertainment, and socializing gratifications; and negatively predicted by information seeking. Fake news sharing is positively predicted by instant news sharing and negatively predicted by fact-checking. The fsQCA revealed three distinct antecedent configurations leading to high sharing among users, demonstrating that sharing is driven by diverse, equifinal pathways rather than a single set of common characteristics. Study 2 confirmed that self-reported sharing intention predicts sharing intention in task scenarios. Gratifications influence users’ fake news sharing through instant sharing and fact-checking, and three configurations prompt users to share fake news. These findings promote the cultural richness of the fake news-sharing research and offer practical implications.

## 1. Introduction

Fake news refers to false content that mimics the format of legitimate news (Duffy et al., 2020). Closely related terms also include disinformation and misinformation. Specifically, misinformation refers to false information that is spread without malicious intent (Wardle & Derakhshan, 2017), whereas disinformation refers to false information deliberately created to deceive or cause harm to a specific entity (Wardle & Derakhshan, 2017). Fake news often intertwines the characteristics of misinformation and disinformation. With the explosive growth of social media users, the spread of misinformation on social media platforms has become alarmingly prevalent and can no longer be ignored. For instance, Xiaohongshu (RedNote), one of China’s mainstream social media platforms, released its 2025 mid-year content moderation report. The platform removed 3.2 million deceptive posts, banned 10,000 accounts with identity fraud, and deleted 600,000 low-quality posts with AI-generated content (AIGC) (China Daily, 2025). Users often struggle to identify false information on social media platforms, leading to its rapid spread online (Sun & Xie, 2024).

Uses and Gratifications Theory (UGT) posits that individuals use media to satisfy their needs, thereby obtaining gratifications (Ruggiero, 2000). *Gratification* refers to a sense of psychological satisfaction or experience obtained by users through active participation in media activities (Stafford et al., 2004). In recent years, Uses and Gratifications theory has been widely applied to explain individuals’ social media usage behaviors. Scholars proposed diverse classification systems for gratifications based on specific contexts. In the fields of information dissemination and social media research, gratifications are categorized into three major dimensions—hedonic, social, and utilitarian gratifications (Gan & Li, 2018; Gan & Wang, 2015; Nijjer & Raj, 2020; Sampat & Raj, 2022). When investigating the impact of gratifications on the continuous usage intention of Chinese WeChat users, Gan and Li (2018) classified gratifications into four categories: hedonic gratification (perceived enjoyment, time-passing), social gratification (social interaction, social presence), utilitarian gratification (self-presentation, information recording, information sharing), and technological gratification (media appeal). To explore the relationship between gratifications and fake news-sharing behavior among Indian social media users, Sampat and Raj (2022) divided user gratifications into three types: hedonic (entertainment, time-passing), social (socializing), and utilitarian (information sharing, information seeking). Drawing upon relevant studies on fake news sharing (Ajina et al., 2024; Apuke & Omar, 2021a; Balakrishnan et al., 2021; Sampat & Raj, 2022), this study categorizes gratifications into three types: hedonic gratification (relating to the fulfillment of hedonic expectations, including time-passing and entertainment), social gratification (associating with the fulfillment of social expectations), and utilitarian gratification (referring to the practical satisfaction users gain from engaging with social media platforms, including information sharing and information seeking). Consistent with previous research, this study employed a structured questionnaire to measure gratification. As noted above, there are three dimensions in total. Each dimension comprises three to five items, and all the items are scored on a 5-point Likert scale.

Gratifications have been found to influence fake news sharing (Apuke & Omar, 2021a; Balakrishnan et al., 2021; Wei et al., 2024). However, discrepancies exist among research findings regarding how different dimensions of gratification may affect fake news-sharing behavior. For instance, Balakrishnan et al. (2021) identified altruism (i.e., sharing with the intent to help others) as the primary motivation driving the spread of fake news, whereas time-passing exhibited no significant effect. In their investigation of the underlying motives for sharing fake news online in Malaysia during the COVID-19 pandemic, the results revealed that altruism, ignorance, and entertainment significantly and positively predicted fake news-sharing behavior, with altruism exerting the strongest influence. Conversely, usability, engagement, time-passing, and fear of missing out (FoMO) demonstrated no significant impact. Collectively, this model accounted for 49.2% of the variance in fake news-sharing behavior.

Adnan et al. (2021) also found that altruistic motivation was the primary predictor of fake news sharing among Pakistani social media users; however, factors such as time-passing, information sharing, socializing, and information seeking also exerted significant effects on fake news-sharing behavior during the pandemic. Conversely, Wei et al. (2024) found that time-passing was a key predictor of fake news sharing among Pakistani university students. Information seeking showed a positive yet nonsignificant effect on fake news sharing. Social networking, entertainment, and information sharing all displayed nonsignificant negative trends. Similarly, Apuke and Omar (2021a) observed that time-passing (along with information sharing, socializing, and information seeking) significantly predicted the sharing of COVID-19-related fake news among Nigerian social media users. In contrast, entertainment showed no significant relationship with fake news sharing.

Concurrently, a limited number of studies have focused on the mechanisms through which gratifications influence fake news sharing. Sampat and Raj (2022) explored how gratifications impact fake news-sharing behavior among Indian social media users within the Stimulus–Organism–Response (S-O-R) framework. Their results revealed that time-passing, information seeking, information sharing, and socializing gratifications significantly and positively predicted instant news sharing. The personality traits of extraversion, neuroticism, and openness also significantly and positively predicted instant news sharing, whereas agreeableness and conscientiousness promoted fact-checking. Furthermore, instant news sharing significantly and positively predicted fake news sharing, while fact-checking significantly and negatively predicted fake news sharing. These findings suggest that the impulse for instant sharing, driven by specific gratifications (particularly information sharing) and personality traits, is a critical mechanism underlying the spread of fake news. Conversely, verification behaviors can effectively reduce its spread. Similarly, Ajina et al. (2024) further used the S-O-R framework to validate how various gratifications (Stimulus) significantly influence the fake news-sharing intentions (Response) of Pakistani social media users through cognitive and affective attitudes (Organism).

Although existing studies have shown some evidence that gratifications affect information sharing behavior, the limited research and inconsistent findings make the conclusion uncertain. Cultural differences among participants may be the primary reason for these inconsistencies. The detection and sharing of fake news can vary across cultures. For instance, a cross-cultural study by Ahmed et al. (2024) encompassing eight countries (e.g., China, the United States, the United Kingdom, and Singapore) revealed that although the illusory truth effect of fake news appears to be universal, it also exhibits cultural differences. Among these countries, Chinese participants demonstrated the highest baseline trust in misinformation. Furthermore, Chan et al. (2026) found regional differences in how analytical thinking and conspiracy thinking impact the ability to discern news accuracy on social media. Analytical thinking only decreased the perceived accuracy of misinformation among residents of the United Kingdom and Hong Kong (China). In contrast, conspiracy thinking only increased the perceived accuracy of misinformation among residents of the United States and the United Kingdom. Currently, research on fake news lacks cultural diversity. Murphy et al. (2023) conducted a scoping review of misinformation-related studies published between 2016 and 2022, revealing that 78.12% of these studies originated from the United States (49.93%) or Europe (28.19%), with other regions contributing very little (e.g., East Asia at 5.53% and Africa at 5.27%). Another scoping review focusing on experimental studies regarding misinformation beliefs reached a similar conclusion (Bryanov & Vziatysheva, 2021). In terms of the relationship between gratifications and fake news sharing, the participants were mainly from Pakistan (Ajina et al., 2024), Malaysia (Balakrishnan et al., 2021), and India (Sampat & Raj, 2022).

More importantly, existing research has paid little attention to the relationship between the *questionnaire-based general willingness to share news* and the *willingness to share in experimental task scenarios*. The majority of findings and conclusions are derived from cross-sectional survey data (Apuke & Omar, 2021a, 2021b; Sampat & Raj, 2022), which assess individuals’ general willingness to share fake news. Few studies have investigated whether self-reported general intentions can effectively predict fake news-sharing intentions in specific task scenarios.

Furthermore, regarding data analysis methodology, the existing literature has mainly relied on linear models such as structural equation modeling (SEM) (Apuke & Omar, 2021a, 2021b; Sampat & Raj, 2022) or linear regression analysis (Adnan et al., 2021). SEM were used to examine the isolated effects of independent variables on fake news sharing. However, fake news sharing is a highly complex psychological behavior driven by distinct, equifinal antecedent configurations rather than simple linear relationships. Although SEM can effectively reveal general linear relationships and the net effects among variables, it is limited in revealing the asymmetric pathways and complex configurations of antecedents that lead to high sharing behavior. Conversely, fuzzy-set qualitative comparative analysis (fsQCA) can compensate for this limitation of SEM. Kaya et al. (2020) proposed that using fsQCA (an asymmetric method) as a complement to partial least squares structural equation modeling (PLS-SEM, a symmetric method) enables researchers to not only observe the overall trends among variables but also examine the holistic interactions among various elements. Kang et al. (2024) also suggested that combining fsQCA and PLS-SEM can provide a more comprehensive causal explanation, thereby overcoming the limitations of employing a single method. Using a mixed-methods approach combining PLS-SEM and fsQCA, Yan et al. (2023) explored the impact of information technology empowerment on customer engagement. In a study on the continuous usage intention of Chinese smart voice assistant users, Kang et al. (2024) initially employed SEM and identified responsiveness as the strongest influencing factor. However, further analysis using fsQCA revealed that the combination of responsiveness, subjective well-being, two-way communication, and psychological ownership constituted the core configuration driving users’ strong continuous usage intentions. Therefore, applying the combination of fsQCA and PLS-SEM to study how gratifications influence fake news-sharing intentions is both feasible and necessary.

In summary, under the Stimulus–Organism–Response (S-O-R) framework, this research recruited Chinese social media users as participants and combined two data analysis methods: PLS-SEM and fsQCA. Study 1 aimed to examine the impact of gratifications on the sharing of fake news and the underlying mechanisms. Subsequently, to verify the ecological validity of the findings from Study 1, Study 2 further examined the predictive validity of self-reported fake news-sharing intentions for fake news-sharing behavior within specific task contexts. This research would clarify the impact of gratifications on the sharing of fake news among Chinese social media users. It would further identify the configurations of antecedents that lead to high levels of fake news sharing. Moreover, by focusing on Chinese social media users, the results would help to generate more universally applicable conclusions about how gratifications influence fake news sharing and its mechanisms.

This study primarily addressed the following three research questions:

1. RQ1: How do gratifications influence fake news sharing among Chinese users?
2. RQ2: What configurations of gratifications drive fake news sharing among Chinese users?
3. RQ3: Can self-reported fake news-sharing intentions predict fake news-sharing behavior within specific task contexts?

## 2. The Stimulus–Organism–Response (S-O-R) Theoretical Framework

The Stimulus–Organism–Response (S-O-R) theoretical framework was initially proposed by Mehrabian and Russell within the field of environmental psychology (Mehrabian & Russell, 1974). This framework comprises three core elements: Stimulus (S), Organism (O), and Response (R). Its central premise is that external stimuli influence an individual’s cognitive and affective evaluations, which in turn prompt the individual to generate positive or negative behavioral responses. Specifically, *Stimulus* represents environmental factors capable of eliciting an individual’s cognitive and affective evaluations. *Organism* refers to the internal process of these cognitive and affective evaluations. *Response* denotes the behavioral outcomes resulting from such evaluations, encompassing both behavioral intentions and actual actions (Kang et al., 2024; Shang et al., 2023).

The S-O-R framework has been widely applied to understand consumer behavior, serving as a robust theoretical tool for explaining how gratifications influence individuals’ behavior. In recent years, the S-O-R framework has also begun to be applied to understand how users’ gratifications, acting as Stimulus factors, influence their fake news-sharing behavior through Organism factors (Ajina et al., 2024; Sampat & Raj, 2022). For example, in their investigation of fake news-sharing behavior, Sampat and Raj (2022) used five gratification dimensions (time-passing, information seeking, entertainment, information sharing, and socializing) and five personality traits (agreeableness, conscientiousness, extraversion, openness, and neuroticism) as Stimulus variables (S). They used instant news sharing and fact-checking as Organism variables (O), and fake news sharing as the Response variable (R). Building upon the research of Sampat and Raj (2022), Ajina et al. (2024) tested and validated a modified Stimulus–Organism–Response (S-O-R) theoretical model. They conceptualized three categories of gratifications (hedonic, social, and utilitarian) as Stimulus variables (S), dual-dimensional attitudes (cognitive and affective) as Organism variables (O), and fake news-sharing intentions as the Response variable (R).

Based on existing research, this study conceptualized gratifications as Stimulus factors, encompassing three dimensions and five elements: utilitarian gratification (information sharing and information seeking), hedonic gratification (time-passing and entertainment), and social gratification (socializing). Furthermore, instant news sharing and fact-checking were designated as Organism variables. Finally, fake news sharing served as the Response variable.

## 3. Conceptual Model and Hypothesis Development

### 3.1. Stimulus and Organism Variables

Time-passing: The motivation underlying social media use during free time to relieve boredom or simply fill time. Research indicates that excessive social media use driven by the desire for time-passing is a core factor contributing to problematic use (i.e., social media addiction) (Kircaburun et al., 2020). This aimless, goal-free browsing state can similarly extend to the domain of information dissemination. Laato et al. (2020) noted that when users treat time-passing as their primary objective in social media use, it leads to purposeless, excessive consumption of social media content. Consequently, under information overload, individuals’ cognitive capacities are depleted, leading to a loss of motivation and energy to verify the authenticity of information. Furthermore, research concerning information dissemination has shown that browsing for time-passing is significantly associated with the sharing of false news (Apuke & Omar, 2021a; Wei et al., 2024). To maintain a relaxed browsing experience, users do not allocate additional cognitive resources to verify the accuracy of information prior to sharing. Apuke and Omar (2021a) noted that when individuals use social media for time-passing, their willingness to adequately verify content before forwarding it decreases. To avoid cognitive burden, they tend to skip the effortful verification steps and forward the content directly.

Based on the preceding discussion, the following hypotheses are proposed:

**H1a.**  *Time-passing is positively associated with instant news sharing.*

**H1b.**  *Time-passing is negatively associated with fact-checking.*

Information seeking: The utilitarian gratification wherein news disseminated on social media provides individuals with the latest and necessary information (Apuke & Omar, 2021a; Sampat & Raj, 2022). In crisis contexts (e.g., during a pandemic), people’s desire for information intensifies, thereby driving a greater pursuit of the timeliness of information. Individuals eager to be the first to share information on social networking platforms are more likely to propagate misinformation, particularly within echo chambers that amplify and reinforce pre-existing beliefs among like-minded users (Duffy et al., 2020). Multiple studies have found an association between information seeking on social media and the dissemination of misinformation (Khan & Idris, 2019; Sampat & Raj, 2022).

Based on the preceding discussion, the following hypotheses are proposed:

**H2a.**  *Information seeking is positively associated with instant news sharing.*

**H2b.**  *Information seeking is negatively associated with fact-checking.*

Entertainment: The gratification of entertainment fundamentally involves individuals using social media for amusement and to relieve emotional tension and anxiety (Lee & Ma, 2012). Multiple studies have found that entertainment is one of the primary motivations for people’s social media use (Büchi et al., 2016; Theocharis & Quintelier, 2016). Users who treat entertainment as their primary objective may have their attention diverted from serious news content (Matthes et al., 2023), thereby reducing their willingness to verify it. Furthermore, humorous posts, tweets, and messages shared on social media serve as a coping mechanism in stressful situations (Islam et al., 2020). Many users utilize shared news for entertainment purposes without verifying its authenticity. Consequently, driven by the high-arousal emotions elicited by humorous or novel news, they engage in instant sharing. The study by Islam et al. (2020) found an association between entertainment usage gratification and fake news-sharing behavior, indicating that entertainment motives significantly increase the likelihood of users sharing unverified information.

Based on the preceding discussion, the following hypotheses are proposed:

**H3a.**  *Entertainment is positively associated with instant news sharing.*

**H3b.**  *Entertainment is negatively associated with fact-checking.*

Information sharing: As a key utilitarian motivation, it encompasses users’ behavior on social networks to distribute information to others (Kircaburun et al., 2020). This gratification has been empirically confirmed as a primary driver propelling the adoption and usage of social media (Gan & Li, 2018). Notably, while social media platforms facilitate the efficient flow of information, they also provide a breeding ground for the rapid spreading of misleading content (McGonagle, 2017). According to a study by Talwar et al. (2020) based on the honeycomb model, users frequently pursue the timeliness of information to position themselves as well-informed individuals within social networks. This motivation to maintain a well-informed status drives them to engage in instant sharing to emphasize speed. Furthermore, the findings of Khan and Idris (2019) indicate that when users perceive themselves as possessing high “information sharing skills,” they are more susceptible to blind overconfidence, resulting in sharing behaviors without verification.

Based on the preceding discussion, the following hypotheses are proposed:

**H4a.**  *Information sharing is positively associated with instant news sharing.*

**H4b.**  *Information sharing is negatively associated with fact-checking.*

Socializing: Gan and Li (2018) defined socializing as a form of social gratification, referring to users’ motivation to engage with social media to establish, maintain, or strengthen interpersonal relationships. Cheung et al. (2015) emphasized that online social interaction is central to sustaining the vitality of virtual communities. Through continuous interaction, users enhance their trust in others and their sense of belonging. Existing research has confirmed that social interaction is a crucial factor in promoting continued use of social media (Cheung et al., 2015; J. C. Wang & Chiang, 2009). The study by Lee and Ma (2012) found a positive correlation between the gratification of social needs and news-sharing behavior. Karnowski et al. (2018) further pointed out that when people share news driven by social motives, their primary objective is to interact with others. Consequently, their attention to the shared news content itself is often significantly reduced. Therefore, driven by such objectives, users may exhibit rapid news-sharing behavior while reducing verification behaviors.

Based on the preceding discussion, the following hypotheses are proposed:

**H5a.**  *Socializing is positively associated with instant news sharing.*

**H5b.**  *Socializing is negatively associated with fact-checking.*

### 3.2. Organism and Response Variables

Within the S-O-R framework of the present study, fake news-sharing intentions serve as the final Response variable. It refers to users’ behavioral tendency to share unverified or misleading information on social media. Prior research indicates that such behavior is not necessarily malicious. It is often the consequence of specific cognitive states (i.e., Organism variables) that lead to users’ failure to effectively discern the authenticity of information during the decision-making process (Sampat & Raj, 2022; Talwar et al., 2019).

Instant news sharing and fake news-sharing intentions: The Social Identity Theory proposed by Tajfel and Turner (2004) can explain why individuals engage in instant news sharing to spread their knowledge to others. This theory posits that belonging to a specific group can enhance individuals’ self-esteem. Such groups endow individuals with a social identity, thereby fostering a sense of social belonging. The desire to be situated within a timely and well-informed social network is a primary motivation driving people’s urgency to share information. However, instant news sharing entails potential risks, as it may unintentionally lead to the spread of misinformation. Furthermore, instant news sharing on social media platforms is associated with users’ instinctive emotion-driven behaviors (Lee & Ma, 2012; C. Wang et al., 2015). Within groups composed mainly of acquaintances, social media users may share news immediately upon receiving it. This is because individuals tend to express their opinions cautiously on social media only in unfamiliar contexts (Talwar et al., 2020). Sharing behavior can significantly affect users’ ability to verify news accuracy, thereby reducing their capacity to discern true from fake news (Epstein et al., 2023). Additionally, Apuke and Omar (2021b) identified an association between fake news-sharing behavior and the gratification of instant information sharing. Their further research revealed that users often share information instantly on their preferred social networking platforms without verifying its accuracy. Users subjectively presume that the information received on social media is accurate and thus spread it without prior verification. Based on the preceding discussion, we posit that when users experience the impulse to share news instantly, it becomes difficult for them to verify the accuracy of the news, thereby increasing the likelihood of sharing fake news.

**H6.**  *Instant news sharing is positively associated with fake news-sharing intentions.*

Fact-checking and fake news-sharing intentions: Fake news misleads the public into believing it is accurate through content that mimics the format of legitimate news. The primary reason social media users unintentionally spread rumors is their limited awareness of fact-checking (Talwar et al., 2019). When users assess the accuracy of information before making sharing decisions, the likelihood of sharing fake news decreases. Furthermore, drawing on the honeycomb model, Talwar et al. (2020) proposed that reputation is a core element of social media interactions. Spreading fake news can severely damage an individual’s reputation in social networks. To avoid this risk, individuals consciously filter the content they share, thereby reducing the sharing of fake news. This conscious filtering behavior also aligns with the core tenets of the Social Exchange Theory proposed by Malinowski (2013). When making behavioral decisions, individuals tend to evaluate the potential rewards and costs associated with that behavior. Therefore, to yield more positive outcomes, individuals need to authenticate the news before sharing it.

Based on the preceding discussion, the following hypothesis is proposed:

**H7.**  *Fact-checking is negatively associated with fake news-sharing intentions.*

**Figure 1:**
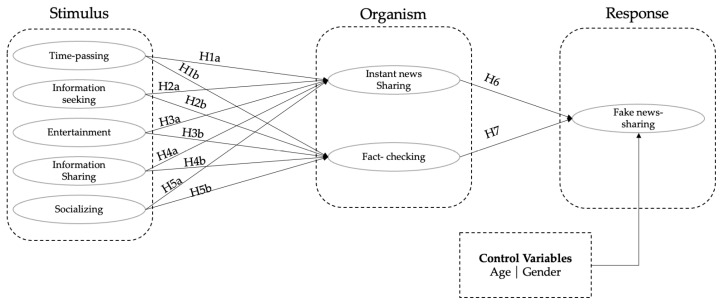
Proposed conceptual model.

The relations mentioned above are presented in the following conceptual diagram depicted in Figure 1.

### 3.3. Self-Reported Fake News-Sharing Intentions and Sharing Behavior in Task Scenarios

Through questionnaire surveys (self-reports), this study measured users’ *general fake news-sharing intentions*. It reflects stable, automated behavioral tendencies during their daily social media use. However, the results are somewhat subjective. In contrast, the *fake news-sharing intentions assessed in the behavioral experiment* constitute a direct observation of individuals’ dissemination willingness in certain task scenarios. To validate the predictive validity of self-reported data concerning data from the experiment, the present study explores the relationship between these two variables.

Prior research indicates that past habits are among the most robust predictors of future behavior (Ouellette & Wood, 1998). Although everyday social media environments differ from behavioral experimental settings, behaviors performed frequently and acquired in stable contexts can predict future behavior through habits, demonstrating cross-situational consistency. In other words, users with a stronger tendency to share fake news may exhibit higher sharing intentions in experimental tasks. Consequently, the sharing tendency measured via self-reports can positively predict fake news-sharing intentions in certain task scenarios.

Based on the preceding discussion, the following hypothesis is proposed:

**H8.**  *Self-reported fake news-sharing intentions are positively associated with fake news-sharing behavior within the task context.*

## 4. Study 1

Drawing on UGT and the S-O-R framework, Study 1 employed PLS-SEM to examine the impact of gratifications on fake news-sharing intentions. Gender and age were included as control variables. Furthermore, the fsQCA was employed to investigate the specific configurations of antecedents that drive fake news-sharing intentions.

### 4.1. Study 1 Materials and Methods

#### 4.1.1. Data Collection

This study employed a survey methodology, using paper-based questionnaires to collect data. Prior to completing the survey, all participants read an informed consent form that outlined the study’s purpose, the principle of anonymity, and their right to withdraw voluntarily. Attention-check questions were embedded in the questionnaire to ensure data quality. A total of 405 questionnaires were distributed and subsequently collected. Ninety questionnaires were excluded because participants failed the attention checks or exhibited abnormal response patterns (e.g., straight-lining). A total of 315 valid questionnaires were retained (78.02%). The final sample comprised 62 males and 253 females, with ages ranging from 17 to 26 years (*M* = 19.45, *SD* = 1.66). All participants were current students enrolled at a university in China.

#### 4.1.2. Measurement Instruments

All measurement instruments employed in this study consist of well-established questionnaires widely utilized in relevant research, with demonstrated reliability and validity. Specifically, the measurement framework was primarily adapted from the questionnaire used by Sampat and Raj (2022) in their study on fake news sharing on social media. To ensure the semantic accuracy and applicability of the original English scales within the Chinese context, a rigorous translation and back-translation procedure was conducted for all English items, supplemented by a small-sample pilot test.

The specific measurement indicators for the variables are detailed as follows:

(1) Stimulus Variables

Uses and Gratifications (UGT): Adapted from the research of Sampat and Raj (2022) and Apuke and Omar (2021a), this construct measures users’ motivations for using social media across five dimensions, comprising a total of 23 items: Time-passing captures the extent to which users use social media to pass the time and alleviate boredom (e.g., “Because it is a habit, just something to do”). Information seeking measures the extent to which users use social media to understand current events and acquire valuable information (e.g., “To keep abreast of current news and events”). Entertainment measures users’ motivations to seek interesting, exciting, or enjoyable content. Information sharing assesses users’ willingness to share opinions, express themselves, or provide useful information to others. Socializing reflects users’ motivations to maintain interpersonal relationships and interact with others through sharing behaviors.

(2) Organism Variables

Instant news sharing: Adapted from the scales by Talwar et al. (2019) and Sampat and Raj (2022), this construct comprises four items designed to measure users’ psychological and behavioral tendencies to immediately forward news information upon exposure, without prior thought or verification (e.g., “I share the news on social networks instantly because I do not have time”).

Fact-checking: Also derived from Talwar et al. (2019) and Sampat and Raj (2022), this variable comprises three items measuring users’ habits of confirming the authenticity of information prior to sharing, such as consulting friends or verifying the source (e.g., “I verify news before sharing it to avoid damaging my social image”).

(3) Response Variable

Fake news-sharing intentions: Adapted from Sampat and Raj (2022), this construct comprises six items measuring users’ intentions to share information in hypothetical scenarios. These scenarios involve news that was later discovered to be a hoax, appeared accurate initially but was later found to be fake, or concerns unverified political, health, or current affairs information.

#### 4.1.3. Study 1 Data Analysis Methods

Data preprocessing was initially conducted, followed by validation of the hypothesized relationships within the structural equation model. Finally, fuzzy-set qualitative comparative analysis (fsQCA) was employed as a supplementary analysis to the SEM results, aiming to identify the configurations of antecedents that lead to high fake news-sharing intentions.

Data Preprocessing: This stage encompassed (1) outlier detection, (2) multicollinearity testing, and (3) common method bias testing.

Structural Equation Model Validation: Following data preprocessing, we used SmartPLS to validate and analyze the PLS-SEM model, adhering to the two-step approach to structural equation modeling proposed by Gerbing (1988) and the model validation component of the six-stage linear framework proposed by Urbach and Ahlemann (2010). First, the measurement model was evaluated by observing the factor loadings of individual items to determine whether they adequately reflect their respective constructs. Reliability analyses were conducted to assess whether the model possesses robust internal consistency reliability, and validity tests were performed to determine whether it exhibits satisfactory discriminant validity. Second, the structural model was evaluated by testing the significance of the paths to ascertain the associations among the constructs.

Fuzzy-set Qualitative Comparative Analysis (fsQCA): This method typically comprises three core steps, data calibration, necessity analysis, and sufficiency analysis, with the first two laying the foundation for the latter.

(1) Data Calibration: To maintain consistency with the PLS-SEM measurement model, this study utilized the latent variable scores generated by SmartPLS 4 as the input data for the fsQCA. The data were subjected to fuzzy-set calibration, transforming the input data into membership scores ranging from 0.0 (full non-membership) to 1.0 (full membership), with 0.5 serving as the crossover point. Furthermore, referencing the percentile thresholds employed in the relevant literature within the same field (Li et al., 2023), namely the 95th, 50th, and 5th percentiles, were established as three qualitative anchors corresponding to full membership, the crossover point, and full non-membership.
(2) Necessity Analysis: In this study, a consistency value exceeding 0.90 was adopted as the criterion for determining necessary conditions (Rihoux & Ragin, 2009). Fake news-sharing intentions were treated as the outcome variable. Seven antecedent conditions (time-passing, information seeking, entertainment, information sharing, socializing, instant news sharing, and fact-checking) were examined to determine whether they constitute necessary conditions for fake news-sharing intentions.
(3) Sufficiency Analysis: Sufficiency analysis employs the truth table algorithm to determine the sufficiency between the configurations formed by the antecedent conditions and the outcome. Given that the sample size exceeded 150, following Fiss’s (2011) recommendation, truth table rows with frequencies below 4 were excluded. Simultaneously, in accordance with the approach of Li et al. (2023), within the truth table algorithm, raw consistency was required to remain above 0.9, and Proportional Reduction in Inconsistency (PRI) consistency was required to remain above 0.7. Ultimately, three solutions were derived: the intermediate, complex, and parsimonious solutions. As the intermediate solution strikes an optimal balance between completeness and interpretability (Rihoux & Ragin, 2009), this study selected it as the core result for interpretation.

### 4.2. Study 1 Results

#### 4.2.1. Data Preprocessing and Descriptive Statistics

Data preprocessing revealed no outliers. Therefore, all data were retained for subsequent analyses. Collinearity diagnostics indicated that all variance inflation factors (VIFs) were below the recommended threshold of 5 proposed by Kock (2015), indicating the absence of multicollinearity in the dataset. Harman’s single-factor test was employed to assess common method bias. A principal component analysis (PCA) extracted eight factors with eigenvalues greater than 1 without rotation. The first factor accounted for 25.74% of the total variance, which fell well below the critical threshold of 40%, indicating that common method bias is not a severe concern in the present study. The descriptive statistics for the data are presented in Table 1.

#### 4.2.2. Measurement Model Validation

In assessing the measurement model, certain items exhibited standardized factor loadings ranging from 0.400 to 0.708. Following the methodological guidelines of Hair et al. (2021), these items were deliberately retained to preserve the content validity and theoretical breadth of the constructs, as their removal would compromise the conceptual integrity of the measurement scales. Reliability was evaluated using composite reliability (CR) and Cronbach’s alpha (CA). As presented in Table 2, the CA coefficients for the latent variables ranged from 0.690 to 0.870. Although the α value for the time-passing construct was 0.690 (slightly below the conventional threshold of 0.70), its CR reached 0.800, a level considered acceptable in exploratory research. Consequently, the measurement model can be deemed to possess robust internal consistency reliability.

Construct validity was assessed via convergent and discriminant validity. The CR for all constructs was above 0.700, with average variance extracted (AVE) values ranging from 0.446 to 0.718. Although the AVE for time-passing (0.446) was slightly below the conventional 0.500 threshold, the convergent validity for this construct was deemed sufficient due to its strong CR value. Regarding discriminant validity, the Fornell–Larcker criterion was employed. Table 2 shows that the square root of the AVE for each construct exceeded its correlations with other constructs. Furthermore, the heterotrait–monotrait (HTMT) ratio (a more sensitive metric than traditional approaches) served as the primary indicator for discriminant validity. As indicated in Table 3, with the exception of the HTMT value between information seeking and socializing (0.859, which is close to the 0.85 cutoff), all construct pairs remained below the rigorous threshold of 0.85. These results suggest that the latent variables in the model are distinct from one another, indicating robust discriminant validity.

#### 4.2.3. Structural Model Validation

In this study, the PLS-SEM algorithm was employed to calculate the path coefficients, and the bootstrapping resampling technique (5000 subsamples, 95% confidence intervals) was utilized to test the significance of the paths. The path testing results are presented in Table 4.

Effect of Gratifications: The results indicated that among the five motivations, time-passing (β = 0.254, *t* = 4.952, *p* < 0.001), entertainment (β = 0.191, *t* = 3.106, *p* = 0.002), and socializing (β = 0.279, *t* = 3.187, *p* = 0.001) exerted significant positive predictive effects on instant news sharing. Conversely, information seeking (β = −0.123, *t* = 2.075, *p* = 0.038) significantly and negatively predicted instant news sharing. Furthermore, entertainment significantly and positively predicted fact-checking (β = 0.182, *t* = 2.420, *p* = 0.016).

Impact of Organism Factors: The core paths of the structural model demonstrated strong robustness. Instant news sharing demonstrated an exceptionally strong positive predictive effect on fake news-sharing intentions (β = 0.479, *t* = 8.685, *p* < 0.001). In contrast, fact-checking exerted a significant negative inhibitory effect on fake news-sharing intentions (β = −0.225, *t* = 3.563, *p* < 0.001).

Analysis of the control variables revealed that the effects of gender (β = 0.016, *p* > 0.05) and age (β = 0.051, *p* > 0.05) on fake news-sharing intentions were not significant.

Regarding the model’s explanatory power, the *R*^2^ values were 0.326 for instant news sharing, 0.097 for fact-checking, and 0.239 for fake news-sharing intentions. *R*^2^ values were contingent upon the specific research domain. Although the values in this study are relatively low, they are comparable to those reported in the field of fake news-sharing (Talwar et al., 2019). In terms of the model’s predictive relevance, the *Q*^2^ values were 0.295 for instant news sharing, 0.058 for fact-checking, and 0.090 for fake news-sharing intentions. Hair et al. (2019) asserted that the primary objective of PLS-SEM is prediction rather than explanation. Consequently, even with insufficient *R*^2^ values indicating relatively weak explanatory power, a *Q*^2^ > 0 demonstrated that the model successfully captured the underlying causal relationships among the constructs, confirming its persuasiveness at the predictive level.

To assess the model’s goodness-of-fit, the Standardized Root Mean Square Residual (SRMR) was calculated. In this study, the SRMR value was 0.078, indicating that the model meets the criteria for an acceptable fit.

#### 4.2.4. fsQCA Results

To complement the findings of PLS-SEM, this study employed fuzzy-set qualitative comparative analysis (fsQCA) to identify the configurations of antecedent conditions for fake news sharing. As shown in Table 5, the results of the necessary condition analysis indicated that no single antecedent condition met the requirement for necessity (i.e., none exceeded 0.9) (Fiss, 2011). It demonstrated that no single dimension of gratification, instant news sharing, or fact-checking acted as a necessary condition for fake news sharing. Therefore, it is imperative to further explore the configurations of antecedent variables that contribute to the sharing of fake news.

As presented in Table 6, the intermediate solution from the truth table analysis identified three configurations that prompted users to share fake news. The overall consistency was 0.893, fully exceeding the acceptable threshold, which indicated that these three configurations were reliable pathways leading to fake news sharing.

Configuration 1 was characterized by high levels of information seeking, entertainment, information sharing, socialization, and instant news sharing, alongside the absence of news authentication (coverage = 0.286, consistency = 0.946). This configuration indicated that when users were highly active on social media, they were strongly aroused by multiple motives. Notably, the presence of instant news sharing and the absence of fact-checking acted as the core conditions, while the other interaction motives served as peripheral conditions. Those results suggest that under the complexity of multiple interactive motives, the strong impulse for instant sharing overwhelmed users’ cognitive defenses, prompting them to engage in fake news sharing based on instinct without fact-checking.

Configuration 2 consisted of the presence of high levels of time-passing, entertainment, information sharing, socialization, instant news sharing, and fact-checking (coverage = 0.326, consistency = 0.907). Users in this configuration exhibited strong hedonic and social orientations, accompanied by a certain degree of authentication before sharing. However, the results revealed that within this interplay of motives, the presence of instant news sharing acted as the sole core condition, playing a highly dominant role. Those results indicate that when users were guided by hedonism and social maintenance, their fact-checking was often merely a formality. Due to the strong inertia of instant sharing, even when users performed verification actions, these actions failed to reduce the spread of fake news effectively.

Configuration 3 was characterized by the presence of high levels of time-passing and information seeking, combined with low levels of entertainment, information sharing, socialization, instant news sharing, and fact-checking (coverage = 0.240, consistency = 0.942). In this pathway, the presence of time-passing and information seeking, coupled with the absence of fact-checking, emerged as the core conditions. Users in this configuration lacked the impulse for social interaction and instant sharing, exhibiting significant self-directed characteristics. These results indicate that users whose sole purpose was to pass the time and passively seek information severely lacked awareness of authentication when facing online information. Consequently, such users were equally prone to inadvertently becoming transmission nodes for fake news.

In summary, the overall solution coverage was 0.472, and the solution consistency was 0.893. Both exceeded the thresholds established by Fiss (2011), demonstrating that these configurations possessed robust explanatory power for fake news sharing.

In summary, Study 1 examined the impact of gratifications on fake news-sharing intentions by employing PLS-SEM. The findings reveal that time-passing, entertainment, and socializing gratifications are significantly and positively associated with instant news sharing; information seeking gratification is significantly and negatively associated with instant news sharing; and entertainment gratification is significantly and positively associated with fact-checking. Furthermore, instant news sharing demonstrates a significant positive association with fake news-sharing intentions. In contrast, fact-checking exhibits a significant negative association with fake news-sharing intentions. Additionally, by employing fsQCA to examine the configurations of antecedent conditions for fake news-sharing intentions, this study identified three configurations that effectively explain fake news-sharing intentions.

## 5. Study 2

Study 2 collected participants’ fake news-sharing behavior within a simulated social media scenario, alongside their self-reported fake news-sharing intentions in a non-task-specific context. The objective of Study 2 is to validate the external and ecological validity of self-reported general fake news-sharing intentions by analyzing the correlation between these variables, thereby providing an empirical basis for the generalizability of Study 1’s findings.

### 5.1. Study 2 Materials and Methods

#### 5.1.1. Participants

A total of 98 university students volunteered to participate in the experiment, comprising 31 males and 67 females. The participants’ ages ranged from 17 to 29 years (*M* = 20.29, *SD* = 1.95). All participants had normal or corrected-to-normal vision and provided written informed consent prior to the experiment.

#### 5.1.2. Experimental Materials and Design

The experimental stimuli consisted of 40 news items, comprising 20 real news items and 20 fake news items. The real news items were sourced from reports published by authoritative media outlets, such as Xinhuanet and People’s Daily Online. Conversely, the fake news items were selected from news explicitly verified as false and fabricated by the China Joint Fact-Checking Platform, the Xinhua News Agency App, and the Tencent Jiaozhen fact-checking platform.

During the formal experiment, each participant first completed two practice trials to familiarize themselves with the experimental procedure. Following the practice phase, participants were presented with 20 news items, comprising 10 real and 10 fake items. To counterbalance order effects, the presentation sequence of these 20 items was pseudo-randomized. After reading each item, participants were asked to rate the likelihood of sharing it on social media. The mean score for each participant’s likelihood of sharing fake news items was calculated and used as the behavioral index of fake news sharing within the task context.

#### 5.1.3. Experimental Procedure

**Figure 2:**
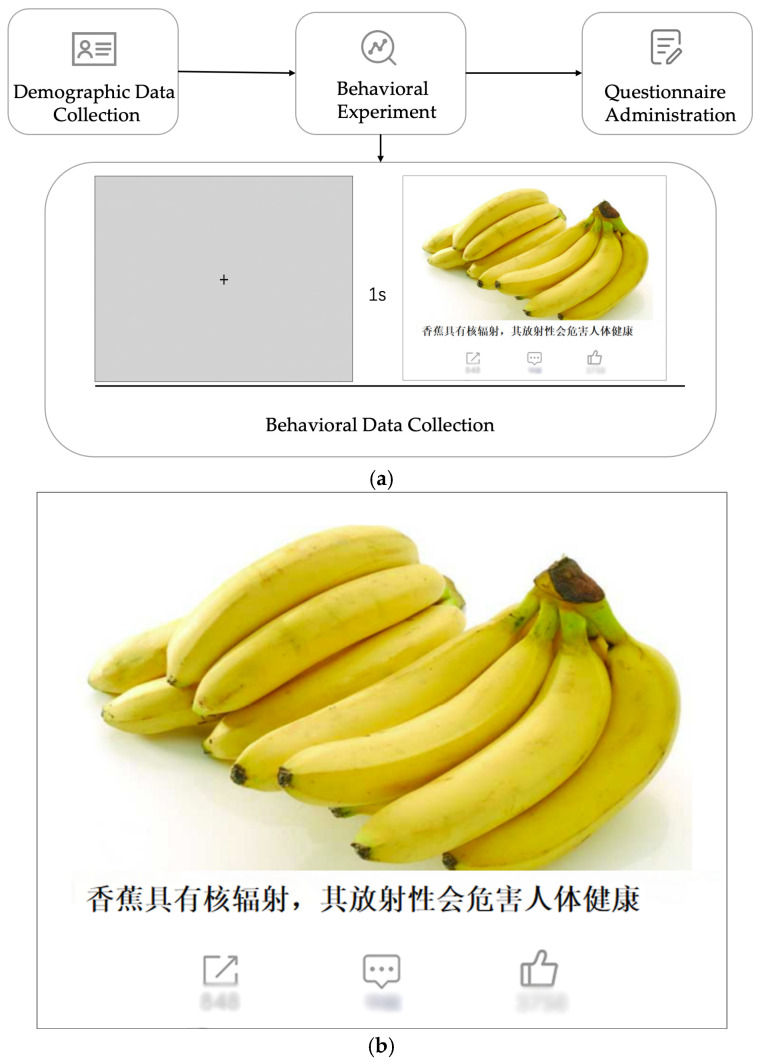
(**a**) Experimental procedure flowchart; (**b**) Example of experimental materials. The three icons are ordered as reposting, commenting, sharing.

The experimental stimuli were presented using E-Prime 2.0 software. Each trial commenced with a fixation cross (“+”) displayed in the center of the screen for 1000 ms, alerting participants that the trial was about to begin. Subsequently, a simulated social media screenshot was presented, and participants were instructed to rate their intention to share the information on a 6-point scale (1 = extremely unlikely to share, 6 = extremely likely to share). Upon a keypress response, the program automatically advanced to the next fixation cross and the subsequent stimulus. The entire experimental task took 10 to 15 min to complete, and all responses were automatically recorded by the computer. Following the behavioral experiment, participants were given a 5 min break for rest and distraction. Then, participants were instructed to complete the questionnaire assessing self-reported fake news-sharing intentions. There were two practice trials to familiarize participants with the experimental procedure. The specific procedure is illustrated in Figure 2.

#### 5.1.4. Study 2 Data Analysis Methods

The statistical analysis for Study 2 aims to determine whether fake news-sharing intentions can predict fake news-sharing behavior within the task context. Given that the behavioral experiment used a single behavioral index, hierarchical linear regression analysis was employed for validation.

First, descriptive statistics and Pearson correlation coefficients were calculated. Subsequently, a two-step hierarchical regression analysis was conducted to introduce the predictor variables incrementally: (1) In Step 1, the control variables were entered (e.g., age and gender). (2) In Step 2, the fake news-sharing intentions variable was incorporated. It was significantly correlated with the fake news-sharing behavior within the task context. Model improvement was evaluated using Δ*R*^2^, F-tests, and standardized regression coefficients (β), with 95% confidence intervals (CIs) reported. All analyses were performed using JASP (version 0.96.0), employing two-tailed tests with a significance level of α = 0.05.

### 5.2. Study 2 Results

#### 5.2.1. Descriptive Statistics and Correlation Analysis

The score of fake news sharing was 3.65 (0.83), and the score of fake news-sharing intentions was 3.36 (0.91). As shown in Table 7, the correlation analysis results revealed a significant correlation between self-reported fake news-sharing intentions and the mean of fake news-sharing behavior within the task context (*p* = 0.03). No significant correlations were observed among other indicators.

#### 5.2.2. Regression Analysis and Hypothesis Testing

Due to the relatively small sample size in Study 2 and the unsuitability of the behavioral data for latent variable modeling, hierarchical regression analysis was employed to validate the predictive role of various variables on the fake news-sharing behavior within the task context. The dependent variable was fake news-sharing behavior within the task context. In the first step, control variables (gender and age) were entered, followed by the inclusion of fake news-sharing intentions in the second step. The results are summarized in Table 8.

Model 1 included only the control variables. The results indicated that demographic variables did not possess significant explanatory power for sharing behavior (*R*^2^ = 0.027, *p* = 0.279).

Model 2 incorporated fake news-sharing intentions into the initial model. The results showed a significant improvement in the model’s explanatory power (*R*^2^ = 0.069, *p* = 0.04). This demonstrates that self-reported sharing intention significantly and positively predicts fake news-sharing behavior in the task context. The above results confirm H8.

In summary, Study 2 confirmed, through hierarchical regression analysis, that participants’ self-reported intentions to share fake news can significantly predict their fake news-sharing behavior in the task context. Although traditional questionnaire surveys offer the scaling advantage of large sample sizes, they are inevitably constrained by self-report bias and subjective limitations. In contrast, situational task experiments can offer more rigorous internal validity, yet they are frequently bottlenecked by high implementation costs and insufficient sample sizes. By cross-validating self-reported and situational behavioral data from the same participant cohort, this study effectively circumvented the limitations of a single method, thereby achieving methodological triangulation. This result confirms the significant predictive validity of users’ self-reported intentions to share fake news for fake news-sharing behavior in experimental task contexts. It also strongly substantiates the robustness, ecological validity, and external validity of the core conclusions drawn in Study 1.

## 6. Conclusions and Discussion

Using PLS-SEM and fsQCA analytical methods, this study systematically uncovered the decision-making processes and configurations of antecedents through which gratifications influence fake news sharing. Furthermore, it investigated the relationship between self-reported fake news-sharing intentions and fake news-sharing behavior within experimental task contexts. The findings of Study 1 revealed that time-passing, entertainment, and socializing gratifications positively influence instant news sharing, whereas information seeking exerts a negative effect. Simultaneously, entertainment gratification positively affects the fact-checking. Regarding the behavioral decision-making process, instant news sharing significantly promotes self-reported fake news-sharing intentions, whereas fact-checking effectively inhibits this tendency. Additionally, the fsQCA analysis revealed three configurations of antecedents that lead to high levels of fake news sharing, confirming the existence of multiple concurrent driving pathways for this behavior. More importantly, the behavioral experiment verified the predictive validity of self-reported fake news-sharing intentions for fake news-sharing behavior within specific task contexts for the first time. Thus, the results in Study 2 provided robust support for the generalizability of the findings from Study 1. Overall, this study not only elucidates the decision-making mechanisms by which gratifications impact fake news-sharing. It also demonstrates that users’ self-reported scale data can effectively predict their sharing decisions in experimental scenarios. In doing so, this study helps bridge the disconnect between “self-reports” and “actual behavior” that has been prevalent in prior research.

### 6.1. Relationships Between Gratifications and Fake News-Sharing Behavior

Regarding the relationship between the Stimulus variables (gratifications) and the Organism variable (instant news sharing), the present results partially support the hypotheses. Specifically, information seeking gratification significantly and negatively predicted instant news sharing, whereas information sharing gratification exhibited no significant association with instant news sharing. Overall, these findings align with existing literature, suggesting that gratifications can influence the behavioral decision-making processes involved in fake news sharing. However, concerning the specific relationships between the various dimensions of gratifications and fake news sharing, the present findings are not entirely consistent with the prior literature. Similarly, within the S-O-R theoretical framework, Sampat and Raj (2022) found that time-passing and socializing gratifications positively predicted instant news sharing among Indian social media users, which aligns with our results. They also discovered that information seeking and information sharing gratifications significantly and positively predicted instant news sharing, while entertainment gratification did not significantly predict INS. Those findings diverged from our results.

Additionally, given that instant news sharing can predict fake news sharing (Apuke & Omar, 2021b; Talwar et al., 2020), it is reasonable to infer that gratifications that influence fake news sharing may also influence instant news sharing. Therefore, the literature examining the relationship between gratifications and fake news sharing provides valuable reference points. However, discrepancies also exist among the findings within those studies. Some findings are consistent with the results of the present study. The relationship between specific gratifications and fake news sharing is well documented, though empirical findings remain highly inconsistent. In alignment with our results, previous research has frequently identified hedonic and social motives (such as entertainment, time-passing, and socializing) as significant positive predictors of fake news dissemination across various demographic groups (Ajina et al., 2024; Apuke & Omar, 2021a, 2021b; Balakrishnan et al., 2021; Wei et al., 2024). Conversely, by highlighting the complexity of this behavior, our findings diverge from certain studies that reported nonsignificant effects for time-passing, entertainment, or socializing gratifications (Ajina et al., 2024; Balakrishnan et al., 2021; Wei et al., 2024). A further divergence concerns information-oriented gratifications. Previous studies have observed that information seeking and information sharing actively promote the spread of fake news (Ajina et al., 2024; Apuke & Omar, 2021a). However, our results align with Wei et al. (2024) regarding information sharing and do not reflect this positive pattern.

Regarding the relationship between the Stimulus variables (gratifications) and the Organism variable (fact-checking), the present results did not support the hypotheses. H1b to H5b examined the negative predictive effects of time-passing, entertainment, information seeking, information sharing, and socializing gratifications on the fact-checking, respectively. Time-passing, information seeking, information sharing, and socializing showed no significant association with news authentication, whereas entertainment significantly and positively predicted it. These results diverge from the existing literature. For instance, Sampat and Raj (2022) discovered that information seeking and entertainment gratifications significantly and negatively predicted fact-checking among Indian social media users.

The results indicate that the impact of gratifications on instant news sharing, fake news sharing, and fact-checking exhibits both cultural universality and cultural differences. The universal pattern lies in the general influence of gratifications on fake news-sharing and verification behavior. At the same time, the cultural specificity is reflected in the differential effects of distinct gratification dimensions on these behaviors. However, as noted in the introduction, empirical evidence remains limited both across and within cultural contexts. Consequently, it is difficult to derive more definitive and directional conclusions regarding how gratifications influence fake news sharing.

Regarding the relationship between the Organism variables and the Response variable (fake news-sharing intentions), the results supported hypotheses H6 and H7. Instant news sharing positively predicted fake news sharing, while fact-checking significantly and negatively predicted fake news sharing. This is consistent with the results of Sampat and Raj (2022), who also discovered that instant news sharing significantly and positively predicted fake news sharing, whereas fact-checking significantly and negatively predicted it. Furthermore, these results support the findings of Talwar et al. (2020) and Apuke and Omar (2021b). Talwar et al. (2020) integrated qualitative and quantitative research methods to explore the specific behavioral manifestations and motivational pathways that lead social media users to share fake news. Their findings revealed that instant sharing behavior driven by the motivation to attract others’ attention had a significant positive impact on fake news sharing. In exploring the motivational factors behind social media users sharing fake news during the COVID-19 pandemic, Apuke and Omar (2021b) similarly found a positive predictive effect of instant news sharing on the sharing of COVID-19 fake news among Nigerian adults. Evidently, research across different cultural backgrounds has yielded similar results, suggesting that the impact of Organism variables on the Response variable may possess cultural universality. However, Talwar et al. (2020) also found that the habit or willingness to authenticate the news before sharing under the influence of time pressure and a sense of moral obligation failed to produce any significant inhibitory effect on reducing fake news sharing. This also indicates that the relationship between the fact-checking and fake news sharing may be partially influenced by temporal contexts or environmental variables.

### 6.2. Complex Antecedent Configurations of Fake News Sharing

PLS-SEM in Study 1 effectively reveals how a single gratification variable linearly influences sharing fake news. However, in real-world contexts, users’ sharing behaviors are rarely triggered by a single form of gratification. Instead, they come from the complex concurrence of multiple motives and organismic factors. Therefore, this study employed fsQCA to conduct a configurational analysis, identifying three sufficient configurations that lead to sharing fake news. Meanwhile, these results are consistent with the conclusions of hypotheses H1a, H2a, H6, and H7 validated by PLS-SEM. These analyses not only achieve cross-method validation but also profoundly reveal the synergistic effects and the “equifinality” mechanism among various factors, substantially extending Study 1.

An in-depth analysis of these three configurations reveals two core psychological evolution pathways. Configurations 1 and 2 share a commonality: both highlight the dominant role of high instant news sharing. However, in terms of their micro-mechanisms, they exhibit fundamentally different cognitive patterns.

Configuration 1 illustrates a sharing logic under “cognitive overload.” When users are highly aroused by multiple motives such as information seeking, entertainment, and socialization, the complex motivational load easily triggers heuristic information processing. In this state, the strong impulse for instant news sharing dominates and bypasses cognitive verification, which is manifested as the concurrent absence of fact-checking. Consequently, individuals rapidly spread false content relying on intuition and instinct.

In contrast, Configuration 2 uncovers a more covert pathway of “confirmation bias.” In this configuration, multiple interactive motives are superimposed with a high-intensity intention to pass time. Although users exhibit a certain degree of fact-checking, the outcome still led to the sharing of fake news. This indicates that under the strong inertia of instant news sharing, such superficial authentication is merely a formality. Driven by strong hedonic and social orientations, users’ cognitive authentication processes are highly susceptible to self-serving bias. The ineffective authentication fails to block the spread of fake news.

Furthermore, Configuration 3 independently reveals a “self-directed” mechanism for SFN. In this configuration, a high level of time-passing and information seeking, with a low level of fact-checking, collectively constitute the core conditions. These results suggest that even if individuals lack the gratifications from interpersonal communication (e.g., low information sharing and low socialization), the transmission of fake news may still be facilitated. As long as there are strong motives of time-passing and information seeking, the sharing behavior could occur. Those findings imply that for users exhibiting “low-involvement” and “lurking” characteristics, aimless browsing and unidirectional information consumption are the norms. Once lacking critical authentication awareness, they could be prone to inadvertently becoming implicit nodes in the spread of fake news.

In conclusion, the fsQCA results not only corroborated the core findings of the PLS-SEM but also deeply elucidated the synergistic interactions and substitution relationships among different gratifications in eliciting aberrant sharing behaviors, thereby providing a robust extension and complement to the linear analysis.

### 6.3. Implications

#### 6.3.1. Theoretical Implications

First, by integrating the Stimulus–Organism–Response (S-O-R) framework and UGT, this study focused on how Chinese social media users engage in fake news sharing. Despite the vast scale of internet and social media use in China, empirical research examining fake news-sharing behaviors remains insufficient. The findings of this study contribute to a more comprehensive understanding of the relationship between gratifications and fake news sharing, shedding light on both cultural universality and cultural specificity within the domain of fake news sharing.

Second, building on conventional PLS-SEM, this study further applied fsQCA to uncover the complex configurations of antecedents driving fake news sharing. The results provide a more comprehensive framework for understanding fake news-sharing behaviors in the real world, marking a novel contribution to the field.

Third, departing from prior research that relies solely on surveys, this study extended beyond questionnaire surveys by designing a behavioral experiment to validate the predictive validity of self-reported fake news-sharing intentions in fake news-sharing behavior within experimental task contexts. Although existing questionnaires have established validity, the findings of this study remain innovative, particularly within the context of fake news sharing. The results provide robust evidence for the generalizability of fake news-sharing questionnaires grounded in the S-O-R framework, contributing to their wider application and the continued accumulation of empirical data on fake news sharing.

#### 6.3.2. Practical Implications

The findings of this study offer the following important practical implications.

First, this study confirmed that time-passing, entertainment, and socializing gratifications drive users to engage in instant news sharing on social media. The tendency to share instant news is positively associated with fake news-sharing behavior. In contrast, the tendency toward fact-checking is negatively associated with fake news-sharing behavior. This study suggests that prior to sharing news driven by hedonic (including time-passing and entertainment) and social motives, social media users should verify the authenticity of information through multiple channels, such as fact-checking platforms. In addition, users should rationally infer the authenticity of the news by synthesizing various cues (e.g., the credibility and professionalism of the news source). Policymakers should launch campaigns promoting news literacy and media awareness to enhance users’ ability to discern the authenticity of information and their discernment in sharing. For users driven by specific gratifications, educational initiatives should cultivate rapid cognitive heuristics, enabling them to develop conditioned, intuitive truth-discernment reflexes during momentary browsing.

Second, the fsQCA identified three configurations that drive users to share misinformation, indicating the complexity of the impact of gratifications on fake news sharing. However, users across all configurations commonly exhibit frequent Instant news sharing or a low willingness for presharing authentication as a core condition. Policymakers should optimize social media algorithmic recommendations, design targeted interventions for instant sharing, and reduce the operational barriers to information verification. These improvements may increase the likelihood of users authenticating the news before sharing and reduce their impulses for instant sharing at the structural and interface levels. Social media platforms can implement ‘friction’ mechanisms in their interface design. For instance, whenever a user clicks ‘share,’ the platform could trigger a confirmation prompt, such as: ‘You have not read the full article. Are you sure you want to share?’ or ‘This information contains controversial content; verification is recommended.’

Finally, government agencies should continuously improve online detection technologies and fact-checking systems for fake news. By promptly correcting fake news on mainstream social media and fact-checking platforms, they may minimize its exposure and ensure that corrective information reaches a wider audience. At the regulatory level, relevant government authorities could establish a standardized, official fact-checking API and mandate its integration across mainstream media platforms. Once high-traffic content is verified as fake news on one platform, this system would immediately trigger cross-platform coordination to proactively restrict its dissemination.

### 6.4. Limitations and Future Research Directions

The present study is subject to several limitations. First, regarding sample representativeness and external validity, data collection primarily relied on convenience sampling, focusing on enrolled university students. Although this demographic represents a high-frequency transmission group for fake news (Zhang et al., 2026), their homogeneous educational and economic backgrounds limit the generalizability of our findings to other age groups (e.g., middle-aged and older adults) or broader social strata. Furthermore, females constituted a larger share of the sample in Study 1, although the current data did not indicate severe gender bias. Future research should employ stratified random sampling to balance gender ratios and incorporate diverse age cohorts to validate the robust applicability of the current findings across different populations.

Second, the current measurement design did not explicitly differentiate users’ truth-discernment capabilities prior to sharing information. Consequently, this study could not strictly distinguish between different behavioral subtypes, such as “blind sharing” (unaware of the falsehood), “warning-oriented sharing” (sharing to alert others), or “hedonic-driven sharing” (knowingly sharing for amusement). Future studies should incorporate truth-discernment assessments in the survey design to untangle the distinct psychological mechanisms and antecedent configurations underlying these three behavioral types.

Third, this study incorporated a behavioral experiment to observe users’ sharing decisions, addressing the limitations of relying solely on self-reported questionnaires. However, the cross-sectional nature of the survey data in Study 1 still restricts rigorous causal inferences regarding the mechanisms linking various gratifications to fake news-sharing behavior. Future research could further validate their dynamic associations through longitudinal designs. Additionally, the current experiment employed static images as stimuli. Compared with the short-video content that currently dominates mainstream platforms, their ecological validity may be constrained. Subsequent experimental designs should focus on investigating the dissemination characteristics of multimodal misinformation in online network environments.

## Figures and Tables

**Table 1:** Descriptive statistics of Study 1.

	Min	Max	*M*	*SD*
Age	17.00	26.00	19.46	1.66
TP	1.00	5.00	3.14	0.72
ISE	1.67	5.00	4.09	0.64
ENT	1.00	5.00	3.28	0.86
ISH	1.00	5.00	3.48	0.83
SOC	1.00	5.00	3.40	0.89
INS	1.00	5.00	2.21	0.90
FC	1.00	5.00	2.62	0.95
FNS	1.00	5.00	2.59	0.93

Note. TP: time-passing; ISE: information seeking; ENT: entertainment; ISH: information sharing; SOC: socializing; INS: instant news sharing; FC: fact-checking; FNS: fake news-sharing intentions.

**Table 2:** Cronbach’s alpha (CA), composite reliability (CR), AVE, and correlations.

Construct	CA	CR	AVE	TP	ISE	ENT	ISH	SOC	INS	FC	FNS
TP	0.690	0.800	0.446	0.668							
ISE	0.707	0.761	0.531	0.274	0.729						
ENT	0.799	0.869	0.626	0.373	0.313	0.791					
ISH	0.859	0.895	0.588	0.291	0.303	0.534	0.767				
SOC	0.870	0.906	0.659	0.276	0.226	0.486	0.740	0.812			
INS	0.814	0.878	0.645	0.389	0.090	0.419	0.414	0.465	0.803		
FC	0.803	0.884	0.717	0.059	0.171	0.263	0.251	0.208	0.205	0.847	
FNS	0.830	0.876	0.543	0.348	0.066	0.166	0.180	0.193	0.433	−0.127	0.737

Note. TP: time-passing; ISE: information seeking; ENT: entertainment; ISH: information sharing; SOC: socializing; INS: instant news sharing; FC: fact-checking; FNS: fake news-sharing intentions. Diagonal elements represent the square root of the average variance extracted (AVE).

**Table 3:** Heterotrait–monotrait ratio.

	TP	ISE	ENT	ISH	SOC	INS	FC	FNS
TP								
ISE	0.418							
ENT	0.489	0.434						
ISH	0.353	0.434	0.631					
SOC	0.344	0.325	0.569	0.859				
INS	0.508	0.118	0.515	0.485	0.549			
FC	0.159	0.164	0.314	0.300	0.25	0.321		
FNS	0.451	0.135	0.206	0.205	0.226	0.524	0.219	

Note. TP: time-passing; ISE: information seeking; ENT: entertainment; ISH: information sharing; SOC: socializing; INS: instant news sharing; ANS: fact-checking; FNS: fake news-sharing intentions.

**Table 4:** Path significance results.

Path		β Value	*t*_Value	*p*_Value	Result
H1a:	TP → INS	0.254	4.952 ***	<0.001	Supported
H1b:	TP → FC	−0.079	1.083	0.279	Not Supported
H2a:	ISE → INS	−0.123	2.075 *	0.038	Supported
H2b:	ISE → FC	0.091	0.741	0.459	Not Supported
H3a:	ENT → INS	0.191	3.106 **	0.002	Supported
H3b:	ENT → FC	0.182	2.42 *	0.016	Supported
H4a:	ISH → INS	0.070	0.813	0.416	Not Supported
H4b:	ISH → FC	0.131	1.389	0.165	Not Supported
H5a:	SOC → INS	0.279	3.187 **	0.001	Supported
H5b:	SOC → FC	0.025	0.291	0.771	Not Supported
H6:	INS → FNS	0.471	8.461 ***	<0.001	Supported
H7:	FC → FNS	−0.212	3.248 **	0.001	Supported

Note. TP: time-passing; ISE: information seeking; ENT: entertainment; ISH: information sharing; SOC: socializing; INS: instant news sharing; FC: fact-checking; FNS: fake news-sharing intentions. * *p* < 0.05, ** *p* < 0.01, *** *p* < 0.001.

**Table 5:** Necessity analysis of individual antecedent conditions.

Conditions	High FNS		Low FNS	
	Consistency	Coverage	Consistency	Coverage
TP	0.742	0.720	0.597	0.609
~TP	0.597	0.585	0.725	0.747
ISE	0.700	0.656	0.673	0.663
~ISE	0.641	0.651	0.651	0.695
ENT	0.700	0.671	0.641	0.645
~ENT	0.629	0.625	0.673	0.702
ISH	0.706	0.678	0.650	0.655
~ISH	0.640	0.635	0.680	0.709
SOC	0.661	0.669	0.617	0.656
~SOC	0.660	0.621	0.689	0.681
INS	0.720	0.749	0.522	0.571
~INS	0.588	0.540	0.771	0.743
FC	0.617	0.621	0.667	0.706
~FC	0.708	0.669	0.642	0.637

Note: ~ represents logical “NOT”, indicating the opposite condition. For instance, “~TP” denotes the absence or low level of time-passing.

**Table 6:** Configurations for the presence and absence of high fake news sharing.

Solution	1	2	3
TP		●	⬤
ISE	●		⬤
ENT	●	●	⊗
ISH	●	●	⊗
SOC	●	●	⊗
INS	⬤	⬤	⊗
FC	⊗	●	⊗
Raw coverage	0.286	0.326	0.239
Unique coverage	0.056	0.105	0.082
Consistency	0.946	0.907	0.942
Solution coverage	0.472		
Solution consistency	0.893		

Notes: ●, ⬤, ⊗, ⊗ indicate the presence of peripheral conditions, the presence of core conditions, the absence of peripheral conditions, and the absence of core conditions, respectively. Blank space indicates “do not care”.

**Table 7:** Correlation analysis of Study 2.

	Age	Sex	FNB	FNS
Age	-			
Sex	0.182	-		
FNB	0.026	−0.153	-	
FNS	0.087	−0.050	0.219 *	-

Note. FNB: Fake news-sharing behavior in task scenarios; FNS: self-report fake news-sharing intentions. * *p* < 0.05.

**Table 8:** Hierarchical regression analysis of fake news-sharing behavior [*B* (*SE*)].

	Step 1	Step 2
Age	0.024 (0.886)	0.015 (0.043)
Sex	−0.291 (0.044)	−0.266 (0.181)
FNS		0.190 (0.091)
	*R*^2^ = 0.027	Δ*R*^2^ = 0.043 *

Note: * *p* < 0.05.

## Data Availability

The data presented in this study are available on request from the corresponding author due to restrictions (privacy, legal or ethical reasons).
